# Regulation of Bone Morphogenetic Protein Receptor Type II Expression by *FMR1*/Fragile X Mental Retardation Protein in Human Granulosa Cells in the Context of Poor Ovarian Response

**DOI:** 10.3390/ijms251910643

**Published:** 2024-10-03

**Authors:** Xuan Phuoc Nguyen, Adriana Vilkaite, Ulrike Bender, Jens E. Dietrich, Katrin Hinderhofer, Thomas Strowitzki, Julia Rehnitz

**Affiliations:** 1 Department of Gynecological Endocrinology and Fertility Disorders, University Women’s Hospital, 69120 Heidelberg, Germany; xuanphuoc.nguyen@med.uni-heidelberg.de (X.P.N.); adriana.vilkaite@med.uni-heidelberg.de (A.V.); ulrike.bender@med.uni-heidelberg.de (U.B.); jens.dietrich@med.uni-heidelberg.de (J.E.D.); thomas.strowitzki@med.uni-heidelberg.de (T.S.); 2 Institute of Human Genetics, University Heidelberg, 69120 Heidelberg, Germany; katrin.hinderhofer@med.uni-heidelberg.de

**Keywords:** FMRP, BMPR2, granulosa cells, poor ovarian response, folliculogenesis, infertility

## Abstract

Fragile X mental retardation protein (FMRP) is a translational repressor encoded by *FMR1*. It targets bone morphogenetic protein receptor type II (BMPR2), which regulates granulosa cell (GC) function and follicle development. However, whether this interaction affects folliculogenesis remains unclear. Therefore, this study investigated the potential effect of FMRP-BMPR2 dysregulation in ovarian reserves and infertility. COV434 cells and patient-derived GCs were used to evaluate FMRP and BMPR2 expression. Similarly, *FMR1*, *BMPR2*, *LIMK1*, and *SMAD* expression were evaluated in GCs with normal (NOR) and poor (POR) ovarian responses. FMRP and BMPR2 were expressed in both cell types. They were co-localized to the nuclear membrane of COV434 cells and cytoplasm of primary GCs. *FMR1* silencing increased the mRNA and protein levels of BMPR2. However, the mRNA levels of *FMR1* and *BMPR2* were significantly lower in the POR group. *FMR1* and *BMPR2* levels were strongly positively correlated in the NOR group but weakly correlated in the POR group. Additionally, *SMAD9* expression was significantly reduced in the POR group. This study highlights the crucial role of *FMR1*/FMRP in the regulation of *BMPR2* expression and its impact on ovarian function. These findings indicate that the disruption of FMRP-BMPR2 interactions may cause poor ovarian responses and infertility.

## 1. Introduction

Folliculogenesis begins with the recruitment of primordial follicles, which later develop into primary, secondary and preantral follicles. Only a subset of antral follicles, the dominant follicles, can reach the preovulatory stage and subsequently ovulate successfully [1]. Paracrine interactions between the oocytes and the surrounding granulosa cells (GCs) take place via locally secreted growth factors and cytokines, which are crucial for normal ovarian function and follicular development [1,2].

The ovarian reserve is critical for the quantity and quality of a woman’s primary follicle pool. A low ovarian reserve (POR) indicates a reduced follicular pool in women of reproductive age and is a common cause of infertility in couples [3]. Fragile X-associated primary ovarian insufficiency (FXPOI) is associated with an expansion of the CGG repeat sequence in the 5′ untranslated region of the X-linked gene FMR1 [4]. Notably, approximately 20% of women with the premutation allele (55–200 unmethylated CGG repeats) are affected by FXPOI [5], which not only leads to subfertility but also increases the risk of conditions linked to early estrogen deficiency. Therefore, to understand the molecular mechanisms of FXPOI, a thorough investigation of ovarian FMR1 mRNA and Fragile X Mental Retardation Protein (FMRP) functions is required [6,7].

At the molecular level, *FMR1* expression levels are elevated in the peripheral blood and brain tissue of premutation carriers [8,9,10]. FMRP, which is encoded by *FMR1*, is reduced in the peripheral blood of FXTAS carrying 140–190 CGG repeats [11]. In addition, FMRP was differentially reduced depending on the length of CGG repeats in the brain and ovarian tissues of *Fmr1* premutation mouse models developed at the National Institutes of Health (NIH) and in the Netherlands [12,13].

FMRP functions as an RNA-binding protein that acts as a translational repressor, suggesting that the dysregulation of its mRNA targets leads to the neuronal and ovarian phenotypes seen in individuals with FMR1 premutation [14,15,16]. Studies have identified the mRNA encoding the type II morphogenetic protein receptor (*BMPR2*) as a target of FMRP [17]. By repressing the *BMPR2* transcript, FMRP suppresses its abundance, but its depletion increases BMPR2 levels. This increase in BMPR2 was notable in the brains of *Fmr1* mutant mice, suggesting that *BMPR2* is an in vivo target of FMRP [17].

BMPR2 is a serine/threonine receptor kinase type II protein. After binding to their receptors, these proteins activate further intracellular signaling cascades through canonical and non-canonical pathways [18,19]. The activation of the type I receptor by BMP ligands triggers the canonical signaling pathway and leads to the phosphorylation of SMAD1, SMAD5, and SMAD9 [19,20]. Additionally, BMPs activate LIM domain kinase 1 (LIMK1) via a non-canonical pathway. LIMK1, which is associated with FMRP, plays a crucial role in actin reorganization and promotes neurite growth and synapse formation [17,21].

BMPR2 is also found in the oocytes and GCs of primordial, primary, secondary, and antral follicles [22]. Growth differentiation factor 9 (GDF9), a BMPR2 ligand, is secreted by oocytes during folliculogenesis [23,24]. It is essential for folliculogenesis and female fertility [25]. The absence of this ligand results in decreased GC proliferation, abnormal oocyte growth, and the failure of follicles to develop beyond the primary stage [25]. GDF9 also inhibits GC apoptosis and follicular atresia [26]. In addition, oocytes regulate proliferation, apoptosis, metabolism, and cumulus cells through the secretion of this paracrine factor [22,24].

This study aimed to investigate the potential impact of FMRP-BMPR2 dysregulation on ovarian reserves and infertility. We hypothesized that FMRP regulates *BMPR2* expression and its downstream signaling pathway in GCs, implying that FMR1/FMRP plays a critical role in folliculogenesis. The present study findings suggest that the dysregulation of this interaction may result in poor ovarian reserves.

## 2. Results

### 2.1. General Study Population

A total of 162 NOR and 35 POR samples were collected for this study. Table 1 summarizes the demographic data of the patients. Significant differences were found in FSH, AFC, AMH, age, total number of oocytes retrieved, and the number of mature MII oocytes. However, no significant differences were found in BMI, estradiol or LH levels.

### 2.2. FMRP, BMPR2, and GDF9 Expression in COV434 Cells and IVF-GCs

**Figure 1:**
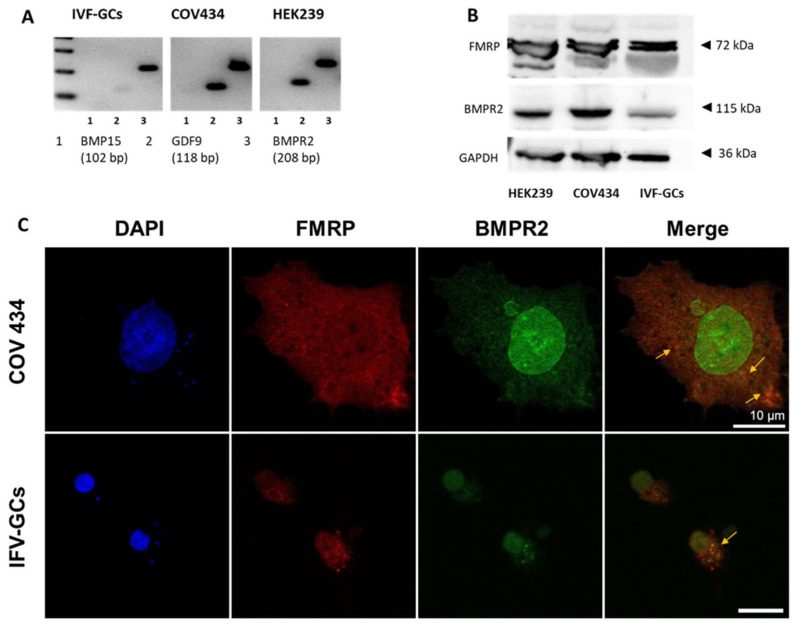
FMRP, BMPR2, and GDF9 expression in COV434 cells and IVF-GCs. (**A**) Total mRNA expression of BMP15 (line 1), GDF9 (line 2), and bone morphogenetic protein type II receptor (BMPR2) (line 3). (**B**) Fragile X mental retardation protein (FMRP) and BMPR2 protein expression in COV434 cells and human granulosa cells (IVF-GCs). HEK 239 cells were used as a positive control. (**C**) Immunofluorescence staining of FMRP and BMPR2 in human GCs. Cells were fixed and stained with FMRP and BMPR2. Cell nuclei were stained with 4′,6-diamidino-2-phenylindole (blue). Alexa 647 (red) and Alexa 488 (green) were used as secondary antibodies for FMRP and BMPR2, respectively. Yellow arrows indicate the colocalization of these proteins in the nuclear and plasma membranes. Scale bar, 5 μm.

PCR and Western blotting were performed to detect the expression of *FMR1*, *BMPR2*, *GDF9*, *BMP15*, FMRP, and BMPR2 in COV434 cells and human GCs (IVF-CGs) obtained from follicular fluid after oocyte retrieval. *BMPR2* mRNA was strongly expressed in both IVF-GCs and COV434 cells, whereas *GDF9* was less strongly expressed in IVF-GCs. In contrast, *BMP15* expression was not detected in either cell type (Figure 1A,B). This indicates that GDF9 and BMP15 are oocyte-secreting proteins, and that their receptor BMPR2 is strongly expressed in GCs. Immunofluorescence staining was performed to determine the localization of FMRP and BMPR2 in COV434 and IVF-GCs. FMRP was co-localized with BMRR2 in the nuclear and plasma membranes (Figure 1C). This expression pattern suggested an interaction between FMRP and BMPR2 in human GCs.

### 2.3. FMRP Regulates BMPR2 Expression in COV434 Cells

**Figure 2:**
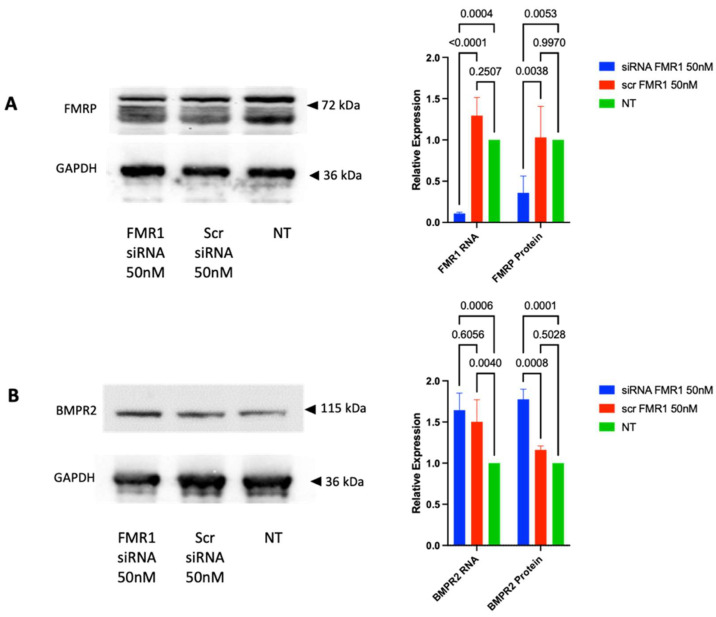
FMRP regulated BMPR2 expression in COV434 cells. Western blotting and qRT-PCR results for *FMR1*/FMRP (**A**) and BMPR2 (**B**) in COV434 cells transfected with *FMR1* siRNA, scrambled siRNA (scr siRNA), or non-transfected cells (NT). Data are expressed as the mean ± standard deviation (SD) (n = 3).

*FMR1* siRNA was knocked down to observe the effect of FMRP on BMPR2 expression in COV434 cells. Transfection of siRNA at a concentration of 50 nM was performed in six-well plates, and cells were harvested after 48 h. The expression of FMRP and BMPR2 was then determined. Our results showed that siRNA treatment led to a significant decrease in FMR1 mRNA and FMRP levels in COV434 cells (Figure 2A). In addition, the decrease in FMRP expression led to a significant increase in BMPR2 mRNA expression (1.64-fold) and BMPR2 protein level (1.77-fold) compared to non-transfected cells (Figure 2B). Our results confirm the regulation of BMPR2 expression by FMRP in human granulosa cells.

### 2.4. FMR1 and BMPR2 Expression in Patients with POR Compared to Those with NOR

**Figure 3:**
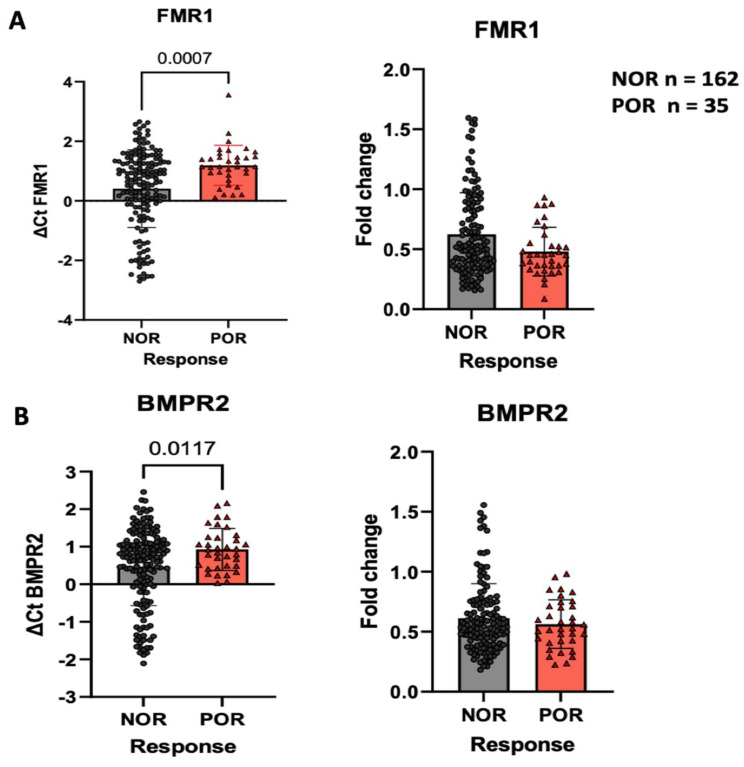
Comparison of FMR1 (**A**) and BMPR2 (**B**) in the GCs of patients with normal (NOR) and poor (POR) ovarian response. The relative mRNA expression levels of *FMR1* and *BMPR2* were significantly lower in the GCs of the POR group than in those of the NOR group. Data are expressed as the mean ± SD.

The expression of *FMR1* mRNA in GCs was significantly lower in the POR group than in the NOR group (Figure 3A) (∆∆Ct: 1.19 ± 0.67, 0.40 ± 1.30, respectively; *p* = 0.0007). Patients with POR also showed significantly lower expression of BMPR2 mRNA than those with NOR (Figure 3B) (∆∆Ct: 0.92 ± 0.56, 0.46 ± 1.03, respectively; *p* = 0.0117). Additionally, *FMR1* and *BMPR2* were expressed in different CGG genotypes (high/low, normal/high, normal/normal, normal/low, and low/low). No significant differences were observed between FMR1 and BMPR2 genotype subgroups (Figure S1).

### 2.5. Expression of BMPR2-Related Signaling Pathways in Patients with POR Compared to Those with NOR

**Figure 4:**
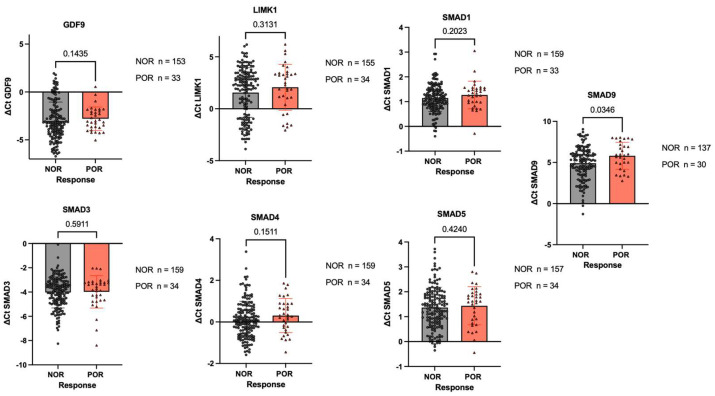
Comparison of BMPR2-related signaling pathways in the GCs of patients with NOR and POR. The relative mRNA expression levels of *GDF9*, *LIMK1*, *SMAD1*, *SMAD3*, *SMAD4*, *SMAD5*, and *SMAD9* in GCs from the NOR and POR groups are shown. Data are expressed as the mean ± SD.

The relative mRNA expression levels of *GDF9*, *LIMK1*, *SMAD1*, *SMAD3*, *SMAD4*, and *SMAD5* were analyzed using TaqMan-based real-time (RT-PCR) in GCs from the NOR and POR groups. As shown in Figure 4, the expression levels of these genes were precisely quantified and compared between the two groups. Significant differences were observed in the expression of *SMAD9*, which was significantly reduced in the POR group compared to the NOR group (*p* = 0.034). This indicates a possible regulatory disruption of downstream BMPR2 signaling pathways in the POR group. 

### 2.6. Correlation between FMR1, BMPR2, and BMPR2-Related Signaling Pathways in the NOR and POR Groups

**Figure 5:**
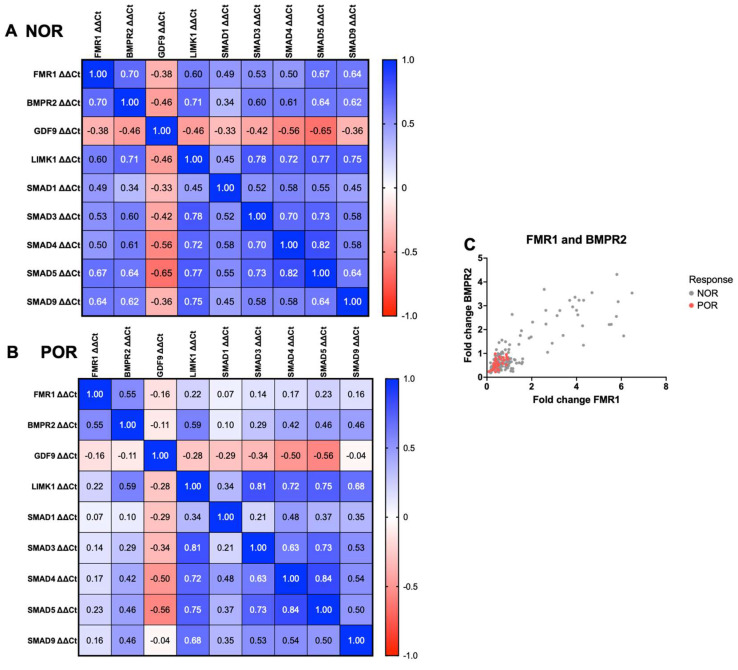
Spearman’s analysis of the correlations of gene expression. Spearman’s analysis was used to evaluate the correlations between the expression of *FMR1*, *BMPR2*, *GDF9*, and *LIMK1*, as well as *SMAD*1, 3, 4, 5, and 9. The correlation matrices for these genes in (**A**) NOR and (**B**) POR are shown. (**C**) Scatter plot of *BMPR2* expression versus *FMR1* expression. The horizontal and vertical axes indicate fold changes in *FMR1* and *BMPR2*, respectively. Interactions between the genes under different ovarian responses are shown.

Spearman’s analysis was used to evaluate correlations between the levels of *FMR1*, *BMPR2*, *GDF9*, *LIMK1*, *SMAD1*, *SMAD3*, *SMAD4*, *SMAD5*, and *SMAD9*. Different correlation patterns were identified between the NOR and POR groups. Several strong positive and negative correlations were observed in the NOR group (Figure 5A), indicating a tightly regulated network of gene interactions. In particular, the expression of *FMR1* strongly correlated with that of *BMPR2* (r = 0.70, *p* < 0.001). In addition, *LIMK1* and several *SMAD* genes showed a positive and statistically significant correlation with *FMR1 mRNA* expression levels (*p* < 0.01 for all parameters). This indicates the possible coregulatory pathways involved in normal ovarian function. The POR group exhibited a different correlation pattern (Figure 5B), with less strong correlations between *FMR1* and *BMPR2* (r = 0.55, *p* < 0.001) and significant correlations between *FMR1* and *SMAD* genes in the NOR group that were previously weaker (*p* < 0.01 for all parameters). This may reflect a disruption in the regulatory network of these genes under POR. Figure 5C shows a scatter plot of *BMPR2* expression versus *FMR1* expression. The clustered data points for the POR group indicate that patients with POR generally have lower expression levels of *FMR1* and *BMPR2* than those with NOR. This clustering indicates a significant downregulation in the expression of these genes in the POR group, which may have contributed to the impaired ovarian response observed in these patients. 

## 3. Discussion

This study provides insight into the regulatory role of FMRP in the expression of BMPR2 in human GCs and its association with POR. Our findings elucidate the molecular processes involved in folliculogenesis and identify pathways that potentially contribute to infertility in women with POR. To our knowledge, this study is the first to confirm the regulation of BMPR2 expression by FMRP in human GCs.

FMRP and BMPR2 were co-expressed in COV434 cells and primary IVF-GCs. The co-localization of these proteins, particularly in the nuclear membrane of COV434 cells and in the cytoplasm of primary granulosa cells, suggests a functional interaction [16,17,27]. This spatial relationship suggests that FMRP directly regulates BMPR2 expression at the translational level. FMRP primarily acts as an RNA-binding protein suppressor of various gene transcripts [28]. Further studies are needed to explore the potential targets of FMRP in ovarian cells, as these could elucidate its role in functional ovarian reserve, ovarian aging, female fertility, IVF outcomes, and autoimmunity. These clinical phenotypes are associated with specific *FMR1* genotypes [5,8,29,30,31].

In vitro experiments demonstrated the suppressive effect of FMRP on BMPR2 expression. *FMR1* silencing in COV434 cells significantly increased the BMPR2 mRNA and protein levels, confirming the repressive effect of FMRP on BMPR2. This regulatory interaction is consistent with the findings of Kashima et al. [17], who showed that FMRP regulates BMPR2 expression by binding to specific FMRP binding motifs (FBMs) in the C-terminal domain sequence (CTDseq) of BMPR2 mRNA, inhibiting it in neuronal cells. This protein particularly affects the full-length isoform that activates LIMK1, a key player in non-canonical BMP signaling. These findings suggested a conserved regulatory mechanism of FMRP on BMPR2 in various cell types, including GCs and neurons. These findings may be applicable to the regulation of BMPR2 by FMRP in oocyte development. Rosario et al. [32] highlighted the role of FMRP in human ovarian development. FMRP was associated with cytoplasmic granules at the onset of meiosis in human oocytes, suggesting a role in mRNA metabolism during crucial maturation processes [32]. Our study highlights the importance of FMRP in BMPR2 regulation, a key signaling pathway during oocyte development and folliculogenesis [23,24,33].

We analyzed the expression and correlation of FMR1 and BMPR2 in women with NOR and POR. The different expression patterns observed in the NOR and POR groups highlight the importance of the interaction between FMRP and BMPR2 in ovarian responses. In particular, mRNA levels of *FMR1* and *BMPR2* were significantly lower in the POR group. This further emphasized the association between decreased gene expression and impaired ovarian response. Moreover, this indicates that an optimal amount of *FMR1*/FMRP elicits an optimal ovarian reserve. Thus, deviations from the norm can lead to an aberrant FMRP amount and impaired functionality, resulting in reduced ovarian capacity. These deviations include the overexpression or under-expression of *FMR1* in the cases of premutation or poor response, respectively, in patients without PM. *FMR1* and *BMPR2* were strongly positively correlated in the NOR group, indicating a tightly regulated gene network. However, these correlations were weaker in the POR group, suggesting a breakdown of the regulatory mechanisms.

The expression levels of FMRP in human GCs warrant further study. However, a limited sample size inhibited the comparison of FMRP expression levels between the POR and NOR groups in the present study. *FMR1* expression negatively correlates with FMRP expression [11,34]. This may be attributed to CGG repeats, as FMRP and FMR1 are negatively and positively correlated with this premutation, respectively. Low FMR1 expression led to increased FMRP levels, which could explain the lower BMPR2 expression observed in the POR group [34,35,36,37]. However, no significant differences were observed between *FMR1* and *BMPR2* expression across CGG repeat genotypes, owing to the small sample size. Therefore, a study with a larger sample size is needed to provide clearer insights.

*FMR1* mRNA may play a dual role in regulating genome organization and gene expression, in addition to its function as a messenger for the production of FMRP [38]. Non-coding RNA produced from protein-coding regions can impact epigenetic mechanisms without translating into protein products. Notable variations in the transcription and expression of FMR1 mRNA could hold biological importance, regardless of their influence on FMRP levels [37]. Moreover, emerging evidence suggests that changes in FMR1 mRNA levels may lead to the dysregulation of miRNAs targeting the 3′UTR, which could further influence FMRP production [39,40,41]. This interaction potentially creates a feedback loop in which changes in FMR1 expression may affect BMPR2, and BMPR2 signaling may in turn modulate FMR1 via miRNA-mediated pathways or other regulatory mechanisms. Changes in mRNA levels or the ratio of the different FMR1 isoforms can influence not only BMPR2 expression but also the expression of steroidogenic enzymes and hormone receptors. These changes may occur through epigenetic modifications or by affecting the translation and cellular distribution of FMRP, potentially leading to ovarian dysfunction and infertility [42,43,44]. Further investigation of these changes could elucidate the contribution of *FMR1* to physiological and premature ovarian aging and female infertility.

Boustanai et al. [42] recently demonstrated that *FMR1* premutation carriers exhibit an impaired ovarian response. This response was attributed to the sequestration of Sam68, an RNA-binding protein involved in the maturation of the FSH receptor (FSHR) transcript. The expanded CGG repeat transcript in FMR1 PM carriers sequesters Sam68, resulting in a reduced amount of free Sam68 available for processing FSHR precursor transcripts. This dysregulation leads to decreased FSHR levels, which contribute to a decreased ovarian response. Our findings are consistent with this research, indicating an impaired amount of *FMR1*/FMRP in poor responders. These findings further suggest that the dysregulation of this interaction may affect other molecular signaling pathways, including those involving Sam68, further impairing ovarian function.

BMPR2 plays a central role in apoptosis and follicle development, which are mediated by the BMP signaling pathway [18,45]. BMPs, which belong to the TGF-β superfamily, are crucial for regulating follicle growth and oocyte quality. BMPR2 activation by BMP ligands in GCs leads to the phosphorylation of receptor-regulated SMADs (such as SMAD1, SMAD5, and SMAD9), which then migrate to the nucleus and regulate the transcription of target genes involved in cell proliferation, differentiation, and apoptosis. This pathway ensures the proper development and maturation of follicles and survival of GCs [20,46,47]. In addition, BMPs inhibit GC apoptosis, promote proliferation, and maintain steroidogenic activity, which are essential for the successful progression of folliculogenesis [18,23,39].

The correlation between *FMR1* and genes of the BMP signaling pathway, including *BMPR2*, *LIMK1*, and *SMADs*, was stronger in the NOR group than in the POR group. Furthermore, the mRNA expression levels of SMAD9 were significantly lower in the GCs of the POR group than in those of the control group, which may be due to reduced BMPR2 expression. These results underscore the critical role of FMR1/FMRP regulation in the BMPR2 signaling pathway. The disruption of this regulatory link may lead to impaired GC function and contribute to POR. Further functional studies, such as on the overexpression of FMRP and suppression of BMPR2 in granulosa cells, are needed to directly establish the link between FMRP-BMPR2 interactions and POR.

**Figure 6:**
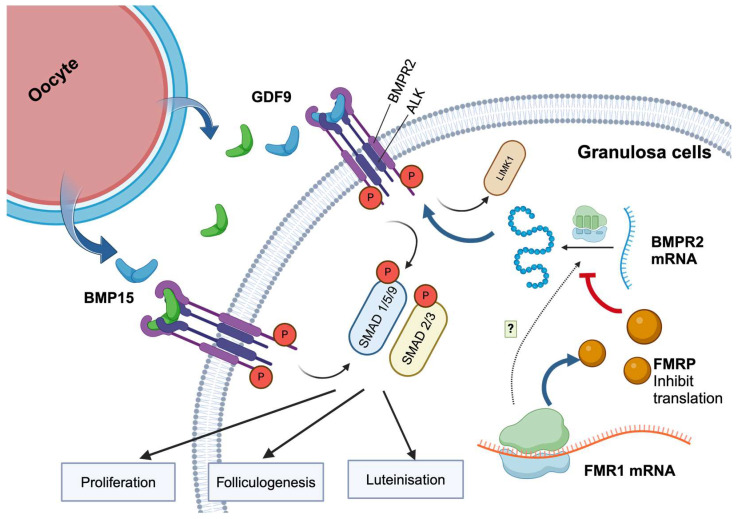
The regulatory role of FMR1/FMRP on BMPR2 in granulosa cells. This figure illustrates the mechanism through which FMRP regulates BMPR2 expression in GCs to affect folliculogenesis, proliferation, and luteinization. BMP15 and GDF9 secreted by oocytes bind to BMPR2 receptors and activate the BMP signaling pathway. This activation leads to the phosphorylation of SMAD1/5/9 and SMAD2/3, which then promotes the expression of genes crucial for GC function. FMRP, which is encoded by the FMR1 mRNA, inhibits BMPR2 mRNA translation, thereby regulating BMPR2 protein levels. Impaired FMR1/FMRP levels in individuals with POR could lead to the disruption of FMRP-BMPR2 interactions, impaired BMP signaling, increased apoptosis, and impaired follicle development. This underscores the critical regulatory role of FMR1/FMRP and its importance in ovarian function and infertility. Figures were created using Biorender (https://www.biorender.com (accessed on 28 August 2024)).

In conclusion, our study highlights the critical role of *FMR1*/FMRP in the regulation of BMPR2 expression and its impact on ovarian function (summarized in Figure 6). These results suggest that the disruption of FMRP-BMPR2 interactions may contribute to poor ovarian response and infertility. Impaired BMP signaling could lead to increased apoptosis and clinical signs of impaired follicular development, proliferation and luteinization via the impaired SMAD pathway and, ultimately, to a poor response Future research should investigate therapeutic interventions targeting this signaling pathway to improve ovarian response and fertility in affected women.

## 4. Materials and Methods

### 4.1. Patients and Ethical Approval

A total of 200 women aged 28–40 years with different ovarian responses were included in this study. Prior to participation, all women gave informed consent and completed a clinical questionnaire. Ethical approval was granted by the local ethics committee of Heidelberg University (approval number: S-602/2013), and the study adhered to the ethical guidelines of the Declaration of Helsinki. The granulosa cells (GCs) were obtained from follicular aspirates during in vitro fertilization (IVF) or intracytoplasmic sperm injection (ICSI) performed between February 2013 and August 2022 at the Women’s Hospital of Heidelberg University, Germany.

According to the ESHRE criteria [48], the participants were divided into two groups: normal ovarian responders (NOR) (n = 162) and poor ovarian responders (POR) (n = 35). Clinical and demographic data were collected including age, body mass index (BMI), baseline hormone levels, and serum concentrations of follicle-stimulating hormone (FSH), luteinizing hormone (LH), estradiol, and anti-Müllerian hormone (AMH). Reproductive parameters such as the number of antral follicle count (AFC), the total number of oocytes and the total number of mature oocytes (MII) were also recorded.

### 4.2. Ovarian Stimulation

Physicians not involved in this study chose either the long gonadotropin-releasing hormone (GnRH) agonist protocol or the GnRH antagonist protocol for ovarian stimulation, as described previously [49]. In the long GnRH agonist protocol, downregulation with a GnRH agonist began on day 21 of the menstrual cycle. Starting on the second day of the next cycle, daily injections of gonadotropins (rFSH, rFSH/rLH or human menopausal gonadotropin [HMG]) were administered to promote follicular growth. Ovulation was triggered with human chorionic gonadotropin when the follicles reached a diameter of 17 mm. The oocytes were then retrieved 36 h later by ultrasound-guided follicular puncture. The retrieved samples were stored in 14 mL tubes containing phosphate-buffered saline (PBS) (ART-4012, SAGE IVF; Cooper Surgical, Trumbull, CT, USA) mixed with heparin (2.5 IU/mL) or a flushing medium (4 GM 501F-100; Gynemed, Lensahn, Germany). In the GnRH antagonist protocol, daily injections of gonadotropins (mainly recombinant FSH or HMG) began on the second day of the cycle to stimulate follicular development. As soon as the leading follicle reached a diameter of 14 mm, a GnRH antagonist was administered to prevent premature ovulation. After ovulation induction, oocytes were retrieved as soon as the follicles reached a diameter of 18 mm. The total dose of gonadotropin was adjusted based on the patient’s individual response.

### 4.3. Retrieval of GCs

Granulosa cells (GCs) were extracted from the follicular fluid after transvaginal ultrasound-guided follicular aspiration for IVF according to previously established protocols [49]. We used 14 mL round-bottom tubes (352001; Falcon, NY City, NY, USA) to collect follicle fluid. Follicle fluid was then added to a cell culture dish (150350 or 150360; Thermo Fisher Scientific, Nunc, Waltham, MA, USA) on a heated bench at 37 °C (Workstation L126 Dual; K-Systems, Birkerød, Denmark). Mural GCs present in the aspirate were identified using a Nikon SMZ1500 zoom stereomicroscope (Nikon Instruments Europe B.V., Amsterdam, The Netherlands). While most GCs were collected directly from the follicular fluid, if blood was present, a brief wash with Multipurpose Handling Medium (MHM; 90163; Irvine Scientific, Santa Ana, CA, USA) with Serum Substitute Supplement (SSS; 99193; Irvine Scientific) or other complete media such as MHM (90166; Fujifilm Irvine Scientific, Santa Ana, CA, USA), Sydney IVF Medium (K-SIFM-20; Cook Medical, Bloomington, IN, USA) or continuous single culture medium (90165; Fujifilm Irvine Scientific) was performed. GCs (2.5 μL) were aspirated using a sterile tip (ep Dualfilter T.I.P.S. 10 μL S; Eppendorf, Wesseling-Berzdorf, Germany) and transferred to 1.5 mL tubes (Sarstedt, Nümbrecht, Germany) prefilled with 12–13 μL RNAlater stabilization solution (Ambion, AM7020; Life Technologies, Carlsbad, CA, USA) and stored at 4 °C.

### 4.4. CGG Repeat Length Analysis

DNA samples were extracted from 10 mL blood samples collected in EDTA tubes using standard methods [49]. The CGG repeat length in the 5′UTR of FMR1 exon 1 was determined by PCR and sequenced using an ALFexpress DNA sequencer (Amersham 1050; Pharmacia Biotech, Freiburg, Germany) or an ABI 3100/3130xl sequencer (Life Technologies/Applied Biosystems, Foster City, CA, USA) [50]. For suspected premutations (PM), Southern blot analysis was performed using a 32P-dCTP radiolabeled p2 probe targeting FMR1 exon 1 CGG repeats. From May 2020, CGG repeat length analysis was performed with triplet repeat primed PCR (TP-PCR) using the AmplideX^®^ PCR/CE FMR1 kit (Asuragen, Austin, TX, USA) according to the manufacturer’s instructions. Fragments were separated on a SeqStudio Genetic Analyzer (Applied Biosystems) and analyzed with GeneMapper (V5, Applied Biosystems). CGG repeat lengths were categorized as low (<26 repeats), normal (26–34 repeats) or high (35–55 repeats) [51]. No high/high genotypes or PM alleles >54 repeats were identified in this cohort.

### 4.5. Gene Expression Analysis

Total RNA was isolated from granulosa cells using TRIzol reagent (Life Technologies) according to the protocol indicated by the manufacturer, using either PEQGOLD PHASETRAP A 1.5 mL (VWR International GmbH, Darmstadt, Germany) or MaXtract high-density 1.5 mL (Qiagen, Germantown, MD, USA) tubes [52]. The mRNA was then reverse transcribed with an oligo-(dT)15 primer and M-MLV reverse transcriptase (RNase H Minus, Point Mutant; Promega, Madison, WI, USA) to synthesize cDNA. Assay kits for FMR1 (Hs00924544_m1), BMPR2 (Hs00176148_m1), GDF9 (Hs03986126_s1), and SMAD1 (Hs00195432_m1) were used for gene expression analysis; SMAD5 (Hs00195437_m1), SMAD9 (Hs00195441_m1), SMAD3 (Hs00969210_m1), and SMAD4 (Hs00929647_m1), together with two housekeeping genes (HPRT and TBP; Hs999909_m1 and Hs00427620_m1) designed by TaqMan, were used by Applied Biosystems (Life Technologies, Foster City, CA, USA). The procedures were performed according to the manufacturer’s guidelines. Samples were analyzed in triplicate under standard PCR conditions using a 7500 Fast Real-Time PCR System (Applied Biosystems, Life Technologies, Foster City, CA, USA). Relative gene expression was determined using the ΔΔCt method [53], with cDNA from COV 434 GCs serving as the calibration standard for each run.

### 4.6. COV434 Cell Culture and Knockdown with FMR1 siRNA

COV434 cells were cultured in Dulbecco’s Modified Eagle Medium (DMEM) supplemented with stable L-glutamine. These cells were maintained in 75 cm^2^ flasks in an environment of 5% CO_2_ at 37 °C prior to transfection. The culture medium was supplemented with 10% fetal calf serum (FCS), 50 μg/mL antibiotic/antimycotic mix, and 1 mM L-asparagine. The transfection mix was prepared on the day of transfection using Opti-MEM specifically for FMR1 siRNA. For each well of a 6-well plate, 50 nM of FMR1 siRNA (cat# 4392420, Silencer Select siRNAs; Thermo Fisher Scientific, Schwerte, Germany) or a control siRNA (cat# 4390843, Silencer Select siRNAs; Thermo Fisher Scientific) was combined with 7.5 μL Lipofectamine 3000 according to the manufacturer’s guidelines. Cells were dissociated, counted to ensure a concentration of 80,000 cells/mL, and then transferred to 1.5 mL Eppendorf tubes and centrifuged at 1500 rpm for 5 min. The resulting cell pellet was resuspended in the transfection reagent and incubated for 20 min at room temperature. The cells were then plated in a 6-well plate containing 1.75 mL of fresh complete medium. Twenty-four hours after transfection, the medium was replaced and the cells were further incubated until needed for RNA and protein extraction.

### 4.7. Immunofluorescence Staining

Human granulosa cells (hGCs) and COV434 cells were immunofluorescently stained according to a previously described protocol [54]. Cells were fixed with 4% formaldehyde for 15 min, permeabilized with 0.2% PBS-Triton X-100 (PBST) for 15 min, and blocked with 4% bovine serum albumin (Life Technologies, Grand Island, NY, USA) for one hour to minimize nonspecific binding. Cells were then incubated overnight at 4 °C with primary antibodies: anti-FMRP (1:500, MA5-15499; Invitrogen, Carlsbad, CA, USA) and anti-BMPR2 (1:500, PA5-21437; Invitrogen). After washing with PBS, secondary fluorescent antibodies (goat anti-rabbit Alexa Fluor Plus 568, A-11011 and goat anti-mouse Alexa Fluor Plus 647, A32728; both 1:1000; Invitrogen) were applied for one hour at room temperature. After three PBS washes, cells were air-dried and mounted with medium containing DAPI (P36931; Invitrogen). EVOS M7000 microscope (Life Technologies, Bothell, WA, USA) was used to capture images.

### 4.8. Western Blotting

Granulosa cells (GCs) were lysed in M-PER™ buffer (Pierce, Rockford, IL, USA) with 150 mmol/L sodium chloride, 10 μg/mL chymostatin, 10 μg/mL antipain, and 1× Halt protease and phosphatase inhibitors (Pierce, Rockford, IL, USA). Lysates were centrifuged at 15,000× *g* for 15 min and protein concentrations were measured using a BCA assay (Pierce, Rockford, IL, USA). We separated 50 ng of proteins on NuPAGE 4–12% Bis-Tris gels (Invitrogen) and transferred to 0.2 μm PVDF membranes (Bio-Rad, Feldkirchen, Germany). The membranes were incubated overnight at 4 °C with primary antibodies: anti-FMRP (1:500, MA5-15499; Invitrogen), anti-BMPR2 (1:500, PA5-21437; Invitrogen), and anti-GAPDH (1:1000, PA1-987; Invitrogen). Detection was performed with SuperSignal West Femto Substrate (Thermo Fisher, Scientific, Rockford, IL, USA) and visualization was carried out with an iBright CL 1500 Imaging System (Thermo Fisher, Singapore).

### 4.9. Statistical Analysis

Data analysis was performed using GraphPad Prism (version 9.3.1; GraphPad Software, Boston, MA, USA), with statistical significance set at *p* < 0.05. The distribution of the data was first determined using the Shapiro–Wilk test. Student’s *t*-test or the Mann–Whitney test was used for a simple comparison between the ovarian reserves (NOR and POR). Correlation was calculated using Spearman–Rho’s correlation coefficient, as not all the genes analyzed were normally distributed. CGG subgroups and an analysis of cohort demographics were compared using one-way analysis of variance or the Kruskal–Wallis test, as appropriate. All data are presented as the mean ± standard deviation.

## Figures and Tables

**Table 1:** Cohort demographics.

	NOR		POR		*p* Value
	n	Mean (SD)	n	Mean (SD)	
Age	162	34.15 (4.16)	35	38.86 (4.56)	<0.0001
BMI	162	23.93 (4.02)	35	23.44 (3.42)	0.503
FSH (U/L)	156	7.84 (2.48)	35	9.81 (3.83)	0.0002
LH (U/L)	161	5.40 (2.58)	35	4.94 (1.95)	0.319
Estradiol (pg/mL)	162	49.82 (29.26)	35	54.96 (20.03)	0.324
AMH (ng/mL)	161	3.12 (2.94)	34	1.07 (0.97)	<0.0001
AFC	95	15.07 (10.88)	22	6.18 (4.09)	0.0003
Total Oocytes	161	11.03 (6.79)	35	4.71 (3.19)	<0.0001
MII Oocytes	105	7.57 (6.19)	24	3.58 (2.60)	0.0025

NOR, normal responder; POR, poor responder; BMI, body mass index; AFC, antral follicle count; FSH, follicle-stimulating hormone; LH, luteinizing hormone; AMH, anti-Müllerian hormone; MII, mature oocytes; and SD, standard deviation. *p* values represent significance levels between NOR and POR.

## Data Availability

Data available from first author on reasonable request.

## Supplementary Materials

The following supporting information can be downloaded at https://www.mdpi.com/article/10.3390/ijms251910643/s1.
