# Supplementary material for: Regulation of Bone Morphogenetic Protein Receptor Type II Expression by FMR1/Fragile X Mental Retardation Protein in Human Granulosa Cells in the Context of Poor Ovarian Response
Source: Int J Mol Sci. 2024 Oct 3;25(19):10643. doi: 10.3390/ijms251910643 (PMC11477111; doi:10.3390/ijms251910643)
Supplement: Supplementary file 1 [file ijms-25-10643-s001.zip › ijms-3214121-supplementary.pdf]

Figure S1

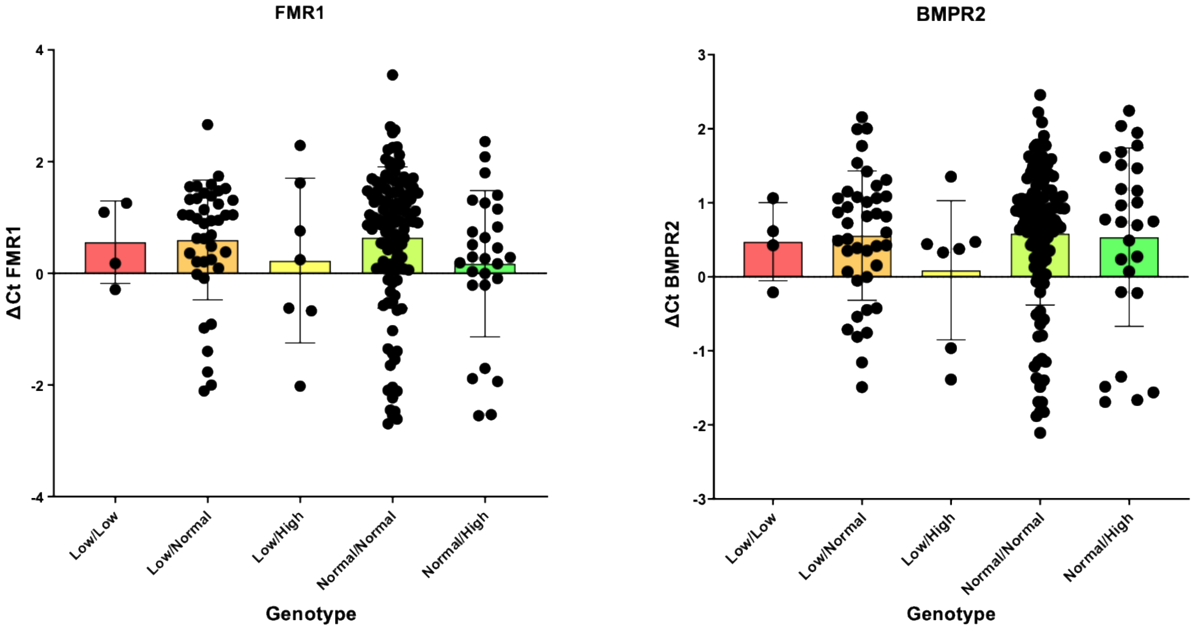

Figure S1. FMR1 and BMPR2 expression in different genotypes.

Expression of *FMR1* and *BMPR2* was observed in different CGG genotypes. Patients were classified into five genotypes according to the repeat lengths of both alleles (low: <26 repeats; normal: 26–34 repeats; high: 35–55 repeats): high/low, normal/high, normal/normal, normal/low, and low/low. No significant differences were observed between the FMR1 and BMPR2 genotype subgroups.
